# Ectopic prostate presenting as a mass in bladder

**DOI:** 10.4103/0970-1591.44270

**Published:** 2008

**Authors:** Filiz Eren, Muhammet Güzelsoy, Bülent Eren, Övgü Aydýn

**Affiliations:** Þevket Yýlmaz Public Hospital, Pathology Department, Bursa, Turkey; 1 Þevket Yýlmaz Public Hospital, Urology Department, Bursa, Turkey; 2 Uludað University Medical Faculty, Forensic Medicine Department, Council of Forensic Medicine of Turkey Bursa, Morgue Department, Ýstanbul, Turkey; 3 Istanbul University Cerrahpaþa Medical Faculty, Pathology Department, Ýstanbul, Turkey

**Keywords:** Bladder, ectopic, prostate

## Abstract

A 24-year-old man presented with dysuria and voiding frequency. Cystoscopy revealed a smooth surfaced nodular mass in the trigonal region. Transurethral insisional biopsy of the mass was done. Histopathological and immunohistochemical examination revealed benign prostatic tissue situated ectopically.

## INTRODUCTION

The term ectopic prostate has been applied to the lesions arising distant from the prostate, although this term has also been used to refer to small foci of prostatic tissue in the region of the bladder trigone and the seminal vesicles.

The finding of ectopic prostatic tissue in the bladder is unusual although there are few case reports in the literature.[1–4] In these reports, the ectopic prostatic tissue was usually found in the bulbar urethra, bladder neck area, or in the region of the trigone.[2,3] The presence of benign prostatic polyps in the prostatic urethra and bladder neck is a common finding. Histologically, two types of lesions were observed, the polypoid and the flat lesions, shared in common a prostatic-type and transitional surface epithelium.[3] However, in the present case the prostatic tissue was ectopically situated in the trigonal region.

## CASE REPORT

A 24-year-old man presented with history of dysuria and voiding frequency of 3 months duration, took antibiotherapy for urinary tract infection. Physical examination did not present abnormality. Hematological and biochemical investigations were within normal limits. Both X-ray and intravenous pyelogram were normal, microscopic hematuria was detected on urine cytologic examination. On the basis of the clinical suspicion of tuberculosis (symptoms and microscopic hematuria) urine acid-fast bacilli (AFB) investigation was performed. Urine examination yielded neither positive AFB smears nor positive AFB culture.

Ultrasonography revealed any properties. Cystoscopy showed a 0.5 × 0.5 cm^2^ solid looking nodular tumoral mass on the midline of the trigonal region. The tumor had smooth surface covered with shiny mucosa. Complete transurethral resection of the tumor was done. Postoperative course was uneventful.

**Figure 1:**
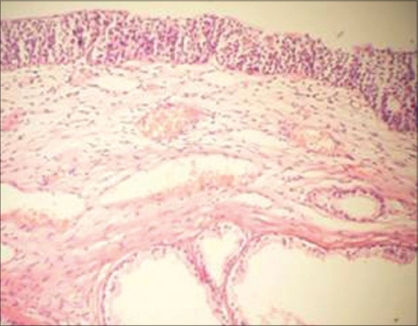
Photomicrograph of the bladder mass showing prostatic glands with secretion in the lumen (H & E, ×100)

**Figure 2:**
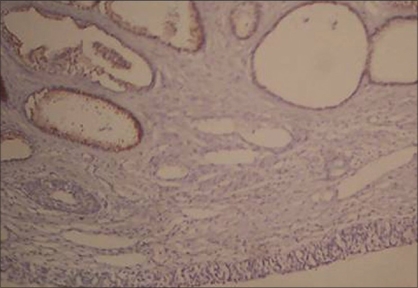
Photomicrograph of the bladder mass showing prostatic glands with PSA positivity (PSA ×100)

Histopathological examination revealed prostatic glands with secretion in the lumen. Immunohistochemical markers study was done to reveal ectopic prostatic tissue, prostate-specific antigen (PSA) and prostatic acid phosphatase (PAP) were applied to paraffin sections using commercially available reagents. Immunohistochemistry staining done on ectopic tissue revealed PSA and PAP positivity. A diagnosis of ectopic prostate was confirmed [Figures 1, 2]. Presently the patient is doing well at a follow-up of 1 year.

## DISCUSSION

Historically, the first evidence of ectopic prostatic tissue was in the form of a group of glands located in the submucosa of the neck of the bladder, which they called the subcervical glandular group. Subsequently other authors also reported this finding.[1,2] The most common site of ectopic prostate is posterior urethra.[2] These occur predominantly in young men and the presenting symptom is usually hematuria and dysuria as in the present case.[2,3]

The other modes of presentations are bladder outlet obstruction and urinary tract infection. Histological, histochemical, and ultrastructural characteristics resemble the prostate.[1,2] In the report of Delladetsima *et al.* histologically, two types of lesions were observed, the polypoid located in various sites of the bladder wall and the flat lesion found in the bladder neck. Both lesions shared in common a prostatic-type and transitional surface epithelium while prostatic-type glands were prominent in the polypoid lesion. Prostatic differentiation of the lesion in the presented case was confirmed by immunohistochemical staining. The glandular cells were strongly positive for PSA and PAP as it was reported in previous researches.[3,5]

On the basis of specific findings, some authors considered the metaplasia as the most reliable histogenetic aspect.[3] Another potential mechanism by which this tissue attained its trigonal ectopic position can be postulated from the normal embryologic origins of the prostate, bladder, and rectum. The bladder and rectum originate from the endodermal cloaca.

A mesodermal proliferation divides cloaca into bladder and rectum, the anterior portion of the bladder further develops into the urethra, which at 3 months gestation extends tubular outgrowths that eventually form the encircling prostate gland. In this case, cells capable of differentiating into prostatic tissue would have traveled with the bladder after division from the bladder, later forming a prostate gland locus.[4,5]

The development of clinically significant prostatic tissue in this area in the postpubertal adults should therefore not be unexpected, albeit rare. The location of the lesion and the presence of vascular congestion correlate well with the clinical findings of dysuria, pollaciuria, and microscopic hematuria. The lesion is histologically and clinically benign. Local persistence or recurrence, however, may result from incomplete removal. There is no report of malignant transformation of ectopic prostatic tissue in the literature.
